# Quality of Life After Stereotactic Body Radiation therapy Versus Video-Assisted Thoracic Surgery in Early stage Non-small Cell Lung Cancer. Is there Enough Data to Make a Recommendation?

**Published:** 2021-04-22

**Authors:** O. Leaman-Alcibar, C. Cigarral, C. Déniz, I. Romero-Palomar, A. Navarro-Martin

**Affiliations:** ^1^Department of Radiation Oncology, Gregorio Marañón General University Hospital, Madrid, Spain; ^2^Department of Radiation Oncology, Salamanca University Hospital, Spain; ^3^Department of Thoracic Surgery. Bellvitge University Hospital. L’Hospitalet (Barcelona), Spain; ^4^Gregorio Marañón General University Hospital Library. Madrid, Spain; ^5^Department of Radiation Oncology, Catalan Institute of Oncology, L’Hospitalet (Barcelona), Spain

**Keywords:** stereotactic body radiation therapy, stereotactic ablative body radiotherapy, video-assisted thoracic surgery, quality of life

## Abstract

**Background and Aim:**

Health reported quality of life test (HRQOLT) in oncologic patients has become a major concern. Early stage in non-small cell lung cancer has two options for treatment in fragile population: Stereotactic body radiation therapy (SBRT) and video-assisted thoracic surgery (VATS). Which option should be recommended in daily clinical practice remains a challenging question. The current review is addressing this concern. Among 1256 articles, 19 met the inclusion criteria and 2034 patients were analyzed treated either with VATS or SBRT. Eleven manuscripts in SBRT, five VATS studies, and three reviews were summarized in the present review. In fragile population, SBRT seems to be a valuable option of treatment with minor or no changes in HRQOLT. However, baseline quality of life status or geriatric assessment tools before treatment could be a good strategy to select appropriate population for undergoing SBRT or surgery.

**Relevance for Patients:**

In this paper, we present a systematic review where we compare the current evidence of two options for treatment in fragile population: SBRT and VATS.

## 1. Introduction

Surgery (lobectomy) remains the mainstay of treatment in early stage non-small cell lung cancer (ES-NSCLC), supported by IB evidence [1]. Nonetheless, patients who do not wish to undergo surgery, or that are not good candidates for surgery due to comorbidities, are often treated with stereotactic body radiation therapy (SBRT) or stereotactic body radiotherapy (IIB evidence) [2].

Efforts have been made to compare SBRT and surgery in early stage operable NSCLC patients; although the lack of accrual and patients; own treatment preferences have made it difficult. However, published data on SBRT inform of a 92% local control at 7 years and 86% regional control [3].

Quality of life (QoL) has become a major concern in oncologic patients. In fact, The American Society of Clinical Oncology recommends since 2015 adding health reported QoL test (HRQOLT) to all clinical trials [4]. In the scenario of early stage NSCLC, at diagnosis 51.5% of patients are 67 years old or older [5]. HRQOLT measures are thought to be fundamental due to the survival in early stage, which is 59% at 5 years [6].

In this manner, some groups suggest that SBRT should become part of the initial treatment algorithm in patients older than 75 years [7] and emphasize the importance of shared decision-making (SDM). So far, advantages related to SDM are reduced costs and improved patient satisfaction [8,9].

Meanwhile, surgical techniques have evolved to become less invasive to reduce postoperative hospital stay and reduce its impact on patients´ QoL [10]. Video-assisted thoracic surgery (VATS) is a minimally invasive surgical technique that has demonstrated similar control rates to that of open surgery and has the advantages of reducing hospital stay and post-operative toxicity [11]. It is not surprising that VATS has become the standard of care in early NSCLC stages and that the vast majority of recent publications on QoL measurements have focused on minimally invasive thoracic surgery.

To the best of our knowledge, an updated systematic review of QoL measures in SBRT versus VATS is needed to understand past biases and design future clinical trials. Therefore, this systematic review tries to elucidate what HRQOLT outcomes are seen in patients diagnosed with early stage NSCLC and treated with either VATS or SBRT. All articles on the topic published until June 2020 were included in the study.

## 2. Search Strategy and Selection Criteria

A systematic literature review was carried out using PUBMED, SCOPUS, and Cochrane databases. Search strategy included the MESH (Medical Subject Heading) terms: SBRT [MESH] OR Surgery, Thoracic [MESH] OR Thoracic Surgery, Video-Assisted [MESH] AND QoL [MESH] AND Lung Neoplasms [MESH]. For original articles, no time frame was established. Review articles on the contrary, had to be published within the past 4 years.

Obtained results from the databases were independently reviewed by four authors (OL), (CC), (CD), and (AN).

Included abstracts for whole text review had to fulfill the next parameters in the box:

- Early stage NSCLC
- Written in English and with complete text available
- Treated either with SBRT or VATS (uniportal, multiportal, or robotic)
- QoL had to be measured at least twice after treatment
- QoL measured by EORTCQLQ-LC13, C30, or SF-36 tests (*)
- Preferably have QoL measures of 6 months or more to measure for chronic toxicity after different treatments and for possible recovery [12,13]

(*) HRQOLT recommended by different associations [14-16].

The European Organization for Research and Treatment of Cancer QoL Core 30 (EORTC QLQ-C30) questionnaire assesses general or global QoL by analyzing 15 items, eight of these items being symptoms that impact in QoL: fatigue, nausea/vomiting, pain, dyspnea, insomnia, appetite loss, constipation, and diarrhea. EORTC Lung Cancer 13 (EORTC QLQ LC-13) questionnaire attempts to provide respiratory-specific measures of QoL summing the scores of symptoms related to lung disease. SF-36; on the other hand, is a HRQOLT that comprises eight domains of health: physical function (PF), physical role (RP), body pain (BP), general health (GH), vitality (VT), social function (SF), emotional role (RE), and mental health (MH).

When review of all abstracts was terminated by four authors, manuscripts that had at least three reviewer’s consensuses were included for the final whole-text evaluation.

**Figure 1:**
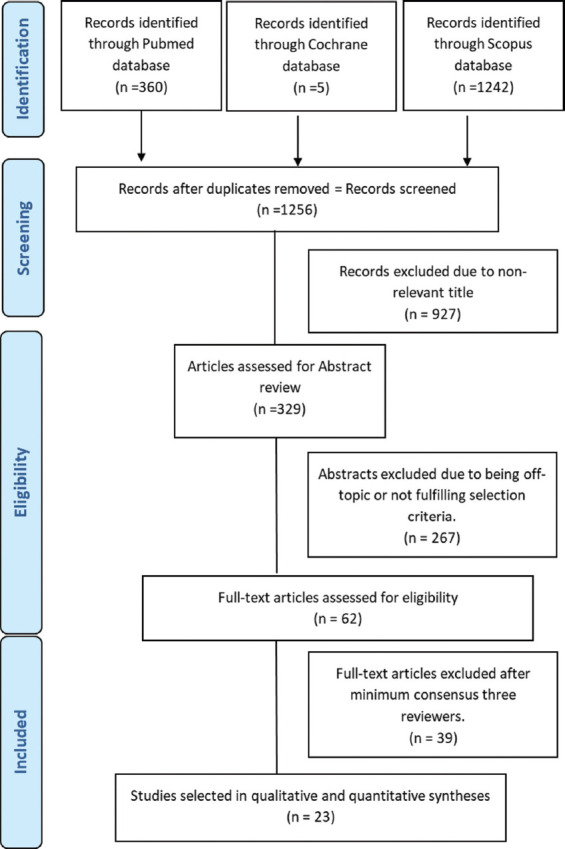
Prisma flow chart

This systematic review has been carried out following the Preferred Reporting Items for Systematic Reviews and Meta-Analyses (PRISMA) guidelines (Figure 1).

## 3. Results

The systematic review carried out using the search strategy explained above gave us as a result 1256 titles after duplicate removal. Title revision reduced the articles to 329, which were then used for abstract review. Selection criteria applied (see Box) on these 329 articles, gave as a result 16 studies and three review articles. Out of the 16 articles, 11 were studies where HRQOLT was measured in patients treated with SBRT, four were studies where HRQOLT was measured in patients treated with VATS and one study was a HRQOLT comparison between SBRT and VATS.

Out of the 11 SBRT studies, nine were prospective observational studies and two were randomized controlled studies, with a total of 1365 patients evaluated. The five VATS studies were four prospective observational studies, and one retrospective study, with a total of 630 patients. In summary, this review included QoL measures of 2034 patients treated either with VATS or SBRT.

### 3.1. QoL in VATS

Selection criteria for the final full-text evaluation on the VATS studies gave as a result four prospective studies and one case review study from the Surveillance, Epidemiology, and End Results Medicare Health Outcomes Survey (SEER-MHOS). A total of 630 patients with a median age of 69.4 are shown in Table 1.

**Table 1:** Surgery studies

Author	Type study	Size	Survey Tool	Assessment time points	Treatment type	Median age	FU	Results	Conclusion
Schwartz *et al*. 2017	Prospective	Surg: 185	SF-12	BL, 1 y	OpS: 100 VATS: 85				
Anami *et al*. 2018.	Prospective	Surg: 36	SF-36	BS, 1 w, 1 m 3 m.	VATS	73.2 y	3 m	PCS: −3.9 (*P*<0.05) at 12 m vs. BS MCS: −2.5 (NS) at }12 m vs. BS RCS: −3 (NS) at 12 m vs. BS	Only significant decrease in PCS.
Avery *et al*. 2020	Prospective	Surg: 110	EORTC QLQ-C30 - EORTC QLQ-LC13. - MFI-20	BS, 1 m, 3 m, 6 m 12 m.	VATS: 92 OpS: 18 Segmental, wedge, lobectomy	69.4 y	12 m	Reduction at 1 month. Increasing at 3 months up to baseline. Reduction in physical role, social function, fatigue and dyspnea not recovered at 12 months
Guang-wen Xu *et al*. 2020	Prospective	Surg: 115	-EORTC QLQ-C30 - EORTC QLQ-LC13	BL, 1 w*, 2 w, 4 w, 8 w	uVATS vs. tVATS Lobectomy	62.3 yo	2 m	Scores non reported. Functional areas decrease after Surg in both treatment modalities. Better in uVATS in functional areas, pain and fatigue score.	uVATS better QOL in short-term
Schwartz *et al*. 2019.	Retrospective Data from SEER-MHOS	SBRT: 28	SF-36 VR-12	BL and 1 y	SBRT	NA 1 y		PCS: −5.6 (95% CI: −9.96, −1.24; *P=*0.0137.	Surg Significant deterioration. SBRT only in PCS.
								MCS: −1.86 (95%CI:−5.4, 1.68; *P=*0.2902)	
		Surg: 156			Surg: SLR or Lobectomy Type of Tech NA		PCS:−4.81 (95%CI: −6.31,−3.30; *P<*0.0001) MCS: −2.96 (95%CI:−4.55,_−1.37; *P=*0.0003)	

SLR: Sublobar resection; PCS Physical Component Summary, MCS Mental Component Summary; FU: Follow UP; BL: Baseline; m; months W: week; VATS: Video-assisted thoracoscopic surgery. uVATS: Uniportal Video-assisted thoracoscopic surgery; tVATS: Three-portal Video-assisted thoracoscopic surgery; OpS: Open Surgery;

* (): Intervention whatever is prior versus post-treatment; NS: Non-significant; SLR: Sublobar resection; NA: Non-available

#### 3.1.1. Prospective studies

In 2017, Schwartz *et al*. [17] published a prospective study comparing open surgery in 100 patients to VATS in 85. QoL was measured before surgery and 1 year later. Employed test was SF12, which is a simplified version of the SF-36 that calculates a physical component score (PCS) and a mental component score (MCS) of the QoL. Results of the study show a statistically significant deterioration in the PF after surgery, whatever this one might be. Multivariate analysis adjusted to age and culture did not modify this deterioration depending on type of surgery.

Anami *et al*. published in 2018 [18] a prospective study in 35 patients with early stage NSCLC treated with VATS and assed for QoL. Its primary objective was to elucidate if prompt physical exercise and rehabilitation could modify QoL scores post-surgery. This physical exercise was taken place only during hospitalization. Items evaluated were muscle force in limbs, resistance to effort, and SF-36 QoL test. Results on the three spheres got worse after surgery, although, physical deterioration persisted at week 12 and was statistically significant in comparison to baseline scores. MCS did not have significant variations and social component, although worsen 1-week after surgery, got back to baseline scores 1-month later. Authors conclude that prompt PF recovery after VATS is possible; however, it is not directly related to a better QoL outcome. This study excluded patients with surgical complications who could not fulfill the physical exercises. QoL scores were likely to be worse in excluded patients.

Avery *et al*. [19] published in 2020, a study on HRQOLT evaluation in 110 patients treated either with open surgery or VATS. In this prospective study, EORTC questionnaires were taken at baseline and in five other time-points during follow-up. Surgery undergone by patients could vary from wedge-type resections, segmentectomy, lobectomy or even pneumonectomy. Results confirmed an important deterioration in all QoL spheres after surgery that would improve at 3 months follow-up. However, a decline in physical, social, and symptoms such as dyspnea and fatigue was detected 1-year post-surgery. Patient characteristics in each surgical group showed a higher percentage of obesity in the pneumonectomy group, as well as earlier tumor stages in the VATS group, which together made the authors conclude these groups were not comparable. To address this caveat, authors designed the VIOLET study (ISRCTN13472721) that is no longer recruiting and is awaiting results.

Finally, Xu *et al*. published in 2020 [20] a prospective study where QoL was evaluated in 115 patients undergoing lobectomy with uniportal versus three-portal VATS. EORTC questionnaires were used and maximum follow-up was 8 weeks. Baseline QoL scores were similar in both groups. Results highlighted how functional scores, overall health status and symptom scales got worse after surgery in all groups. Although gradual recovery was detected during follow-up, baseline scores were not reached. Interestingly, uniportal VATS had better overall scores compared to three-portal VATS, and these differences were statistically significant in functional areas, overall health status and in symptoms such as fatigue and pain (*P* < 0.05).

#### 3.1.2. Retrospective studies

Schwartz *et al*. [21] published in 2019 a case review series from the SEER-MHOS database. The hypothesis was that sublobar resection (SLR) could represent a minimum deterioration on QoL that could be akin to that of SBRT. This hypothesis was based on the previous studies by the same author where HRQOLT data on lobectomy versus SLR favored the latter. Data extracted from SEER database accounted for 184 patients (28 treated with SBRT, and 156 with surgery –26 of them were SLR) from 1998 to 2014. Two time-points were registered: one at baseline and a follow-up survey at maximum 2 years post-treatment. SF-36 was used up until 2006 and VR-12 questionnaires were used from then after. Patients in the SBRT group were older and were more likely to suffer from COPD, emphysema, asthma, or angina. Baseline PCS and MCS scores were significantly higher in the surgical group (PCS *P* = 0.0061 and MCS *P* = 0.0056). PCS deteriorated in both groups after treatment, but MCS deteriorated significantly only in the surgical group; (−2.96 [95% CI: −4.55, −1.37; *P* = 0.0003] for surgery vs. −1.86 [95% CI: −5.4, 1.68; *P* = 0.2902]) for SBRT. A propensity matched analysis was undergone where 22 patients in each group were evaluated. In this case, PCS and MCS changes between groups were no longer significant.

When type of surgery (lobectomy versus SRL) was compared, PCS score deterioration was detected in both groups but MCS scores on the contrary, deteriorated significantly only in the lobectomy patients (−3.11, 95% CI: −4.74, −1.48; *P* = 0.0002). Finally, the SLR (26p) surgical subgroup was compared to the SBRT group (28p). Patient characteristics were slightly different between the groups. For instance, SBRT group had more patients with COPD, diabetes, and higher percentages of coronary events. Results obtained on QoL between SRL and SBRT showed both PCS and MCS general deterioration; although statistical significance was not reached for MCS decline. Authors conclude that there are no QoL differences between patients treated with SRL and SBRT.

### 3.2. QoL in SBRT

Selection criteria for the final full-text evaluation on the SBRT studies gave as a result 12 studies where QoL was evaluated. These studies summed a total of 1178 patients with a median age of 75, 15 (range 65–77) and a median follow-up time of 23 months (range 12–41) Table 2.

**Table 2:** SBRT studies

Author	Type Study	Size	Survey Tool	Assessment Time Points	Treatment Type	Median Age (y)	Median FU (mo)	Results	Comments
Rutkowski *et al*. (Poland),2017	PO	SBRT: 51	EORTC QLQ-C30, LC-13, HAD	BL, 2 wk, 3 mo	NA	74	NA	No detrimental changes in QOL or HAD. GH: ↑5.5% (*P* = 0.025) PF: ↑ 7% (*P* = 0.032) EF: ↑10% (*P* = 0.0003) Insomnia: ↓16% (*P* = 0.003) Anxiety:↓1.65%*/Depression:↓1.66%* Best improvement in COPD(-) patients	
Mathieu *et al*. (Canada), 2015	PO	SBRT: 45	EORTC QLQ-C30, LC-13	BL, end of treatment, 2, 6, 12 mo, then once per year.	60/3, 50/4-5	77	41	No significant changes in QoL scores over time. Trend in EF improvement at 36 mo (14 ± 24%) and minor cough at 30 mo (13 ± 17%) and at 36 mo (13 ± 22%). Transient declines in SF at 12 mo (12 ± 29%) and at 24 mo (11 ± 29%).	Biopsy-confirmed ES-NSCLC non-surgical or refusing surgery (16%)
Ubels *et al*. (Netherlands), 2015	PO	SBRT: 39	EORTC QLQ-C30, LC-30	BL, 3 wk, 2, 4, 6, 9, 12, 15, 18, 21, 24 mo, then every 6 mo until 5y, PR or ꝉ	60/3, 48-50/5-6, 45/3	77	38	GH fluctuated but remained at baseline. PF, RF and CF: improved slowly* Dyspnea: ↑* (score of 17 at 5y) for the data of QLQ-C30 but it was not significant for the QLQ-LC13. Fatigue: increased over time (*P* = 0.05)	Biopsy-confirmed ES-NSCLC non-surgical or refusing surgery (15%)
Wolff *et al*. (Netherlands), 2018	PO. Propensity score matching	SBRT: 261 (41 matched patients to 41 surgical patients)	EORTC QLQ-C30, EQ-5D	BL, 3, 6, and 12 mo	60/3, 54/3, 60/5, 60/8	SBRT: 69.8	12	Baseline: younger patients and lower ECOG for surgical patients (*P*<0.001) No significant changes in overall health utility/QoL among SBRT and surgery after 12 mo. Difference in health utility between ECOG 0 and ECOG 1-2.	
Alberts *et al*. (Netherlands), 2019			EORTC QLQ-C30 y LC-13			Surgery: 66.7		
Jain *et al*. (Canada), 2013	RCT	SBRT: 51	EORTC QLQ-C30, EORTC QLQ-LC13	BL, end of treatment, 1 and 4 mo	52/4, 48/4 (delivered on 4 d vs. 11 d)	74	NA	Baseline: respiratory symptoms (coughing, dyspnea and fatigue) were worse in the 11 d group. Patients in the short-course arm (4 d) had significant* worse scores for dyspnea and PF at 4 mo.	Lung metastasis in 4p
Lagerwaard *et al*. (Netherlands), 2012	PO	SBRT: 382	EORTC QLQ-C30	BL, 3, 6, 12, 18, and 24 mo	60/3, 60/5, 60/8	74	23	Baseline: lowest functional scores for GH, PF and RF. Highest symptom scores for dyspnea, fatigue and insomnia. Significant deterioration in PF scores over time* Baseline physical functioning scores, comorbidity scores, and forced expiratory volume in 1 s correlated with overall survival	15,4%p refusing surgery
(Netherlands), 2010	PO	SBRT: 39	EORTC QLQ-C30, EORTC QLQ-LC13	BL, 3 wk, 2, 4, 6, 9, and 12 mo	60/3, 48-50/5-6, 45/3	77	17	No significant changes in QoL scores over time except an improvement in emotional functioning score (*P*=0.02)	Biopsy-confirmed ES-NSCLC non-surgical or refusing surgery (15%)
Louie *et al*. (Netherlands), 2015	RCT. ROSEL Trial. Stage I NSCLC: SBRT vs. Surgery	SBRT: 11	EORTC QLQ-C30, LC-13, EQ-5D	BL, 3, 6, 12, 18, and 24 mo	SBRT: 54/3, 60/5	65	SBRT: 40.2	GH events: 8 surgery vs. 2 SABR (HR 1 vs*.* 0.19, *P=*0.038). No other significant changes.	small sample size. Early closure due to lack of recruitment
		Surgery: 11					Surgery: 35.4		
Widder *et al*. (Netherlands), 2011	PO	SBRT arm: 202	EORTC QLQ-C30, EORTC QLQ-LC13	3, 6, 12, 18, and 24 mo	SBRT: 60/3, 60/5, 60/8	76	13	No changes for Global QOL and PF except for patients with high CCI (>3) with a decreased in PF (*P*=0.02) Dyspnea: significative↑3.2 (95% CI: 1.0–5.3; *P<*0.01). Compared with SBRT → 3D-CRT group↓in PF (*P*<0.01) with trend to increase in dyspnea	
		3D-CRT arm: 27			3D-CRT: 70/35	71			
Adebahr *et al*. (Germany), 2018 Nestle *et al*. (Germany), 2020	PO	SBRT: 97 (complete FU: 80)	EORTC QLQ-C30 EORTC QLQ-C30 QLQ-LC13	Bl, 2, 7 wk, 3, 6, 9, 12, 15, 18, 21, and 24 mo	37.5/3, 35/5 (prescribed 60% isodose of PTV)	72	28,6	In short (7 wk) and Long-term FU (2 y): stable QoL/GHS, functions-scores and symptoms. For QoL/GHS, poor baseline QoL/GHS scores (<50) → better significantly improvement (*P*<0.001)	ES-NSCLC non-surgical: 56; ≤2 lung metastases: 44

BL=Baseline. D=Days. Wk=Weeks. Mo=Months. Y=Years. *P=*Patients. ES-NSCLC: Early-stage non-small cell lung cancer. GH: Global health. PF: Physical functioning. RF: Role functioning. CF: Cognitive functioning. EF: Emotional functioning. SF: Social functioning. EORTC QLQ-C30: European Organization for Research and Treatment of Cancer Quality of Life – Core Questionnaire. EORTC QLQ-C30: European Organization for Research and Treatment of Cancer Quality of Life – Lung Cancer Questionnaire. FACT-L: Functional Assessment of Cancer Therapy-Lung. EQ-ED: European Quality of Live Five-Dimension. LCSS: Lung Cancer Symptoms Scale. HAD: Hospital Anxiety and Depression Scale. UCSD-SOBQ: University of California at San Diego Medical Center Pulmonary Rehabilitation Program Shortness-of-Breath Questionnaire.

* statistically significant changes at *P<*0.05. PO: Prospective observational study. PR: Progression. ꝉ: Death

All the included studies were prospective in design. two out of 12 were randomized, one of which compared QoL with surgical patients [22] and the other compared QoL in two different SBRT schemes (4-day treatment versus 11-day treatment) [23]. One study [24,25] carried out a comparison between surgical patients and SBRT using the propensity score matching statistical tool to estimate the effect of the treatments avoiding selection bias. Eight of the studies [22,23,26-31] included, as well as the statistical significance on QLQ tests, a minimum threshold of at least 10 point difference to consider changes that were clinically meaningful [1]. Ultimately, two of these studies were updates of other studies already published [25,31].

Rutkowski *et al*. [32] published a prospective study where 51 patients treated with SBRT were tested for QoL measures through EORTC QLQ-C30 and LC-13 questionnaires. In addition, their level of anxiety and depression with HAD scales was evaluated. They concluded that SBRT did not have a deleterious effect on QoL and psychological functioning reporting a significant improvement in physical functioning (↑7%, *P* = 0.032) and in emotional functioning (↑10%, *P* = 0.0003) at 3 months, with a significant decreased severity of insomnia (↓16%, *P* = 0.003). The greater improvement was observed among patients without chronic obstructive pulmonary disease (COPD). A significant correlation with anxiety and depression was also described for global health, physical, and emotional functioning, and the level of insomnia.

Mathieu *et al*. [26] reported favorable long-term QoL and pulmonary function in 45 biopsy-confirmed NSCLC patients treated with SBRT. A 10-point change from baseline on the 100-point scale was considered clinically significant. The worst baseline functional scores were for global QoL and physical functioning and for dyspnea and coughing symptoms. QLQ-LC13 data evidenced a trend to improve on the emotional score at 36 months (14 ± 24%) as well as for coughing symptom with a reduction of 13 ± 17% and 13 ± 22% at 30 and 36 months, respectively.

Ubels *et al*. [33] prospectively evaluated 39 histological confirmed NSCLC for 5 years. Global health status increased during the first 1.5 years to a score of 4 but decreased to baseline point at the end of the follow-up. The physical, role and cognitive functioning significantly improved slowly over time (*P* = 0.004). The emotional functioning score improved significantly in the 1^st^ year (*P* = 0.0003) but declined thereafter. Respiratory symptoms such as dyspnea and coughing had initial fluctuations with a slow deterioration starting the 2^nd^ year. These symptoms were statistically significant for dyspnea in the QLQ-C30 (*P* = 0.0006) but not in the QLQ-LC13 test. In the same manner, fatigue punctuation worsened with time (*P* = 0.05).

Wolff *et al*. [24] published a prospective study in 2018 with two databases of patients diagnosed with early stage NSCLC: one group treated with SBRT (261 patients) and the other group treated with surgery (41 patients). Primary objective was health utility differences between groups and propensity score matching was used to adjust for possible selection biases. Surgery was either thoracotomy (87.8%) or VATS (12.2%). SBRT, on the other hand, was administered in 3 to 8 fractions with a BED > 100Gy. Patient characteristics analysis showed that the surgical group had younger patients (66.7 years old vs. 69.8) and a better ECOG status. To measure health utility, European Quality of Live Five-Dimension (EQ-5D) (mobility, self-care, usual activities, pain/discomfort, and anxiety/depression) test was used, which is based on items of the QLQ-C30 test. A minimum difference of 0.07 had to be seen in the algorithm to detect differences between both groups. Posteriorly, in 2019, Alberts *et al*. [25] published the results of the same patients with a longer follow-up (1 year). In both cases, long-term differences did not reach statistical significance.

In 2013, Jain *et al*. [23] conducted a prospective study in 51 patients treated with SBRT randomized to receive 48Gy-52Gy in 4 fractions on 4 days versus 11 days. Questionnaires used to evaluate QoL were EORTC QLQ-C30 and QLQ-LC13. Data were collected at baseline, the last day of treatment, 1- and 4-months post-treatment. The proportion of patients with ≥ 10-point change from baseline was considered a clinically significant result. The group treated in the 11-day schedule had worse basal respiratory symptoms (cough, dyspnea, and fatigue) (*P* = 0.02), and in contrast, it was the group with the best outcomes in dyspnea at 1 and 4 months (44.4% vs. 15.4%, *P* = 0.02; 38.5% vs. 12%, *P* = 0.03, respectively). In addition, it was the group with a better physical functioning at 4 months (46.2% vs. 16%, *P* = 0.02). There were no differences found in the rest of the parameters evaluated.

One of the largest prospective studies was published in 2012 by Lagerwaard *et al*. [27]. It included 382 patients diagnosed with early stage NSCLC and who refused surgery (15.4%) or were medically inoperable (84.6%). In this study, risk-adapted fractionation (60 Gy in 3, 5, or 8 fractions) was administered depending on tumor location (central or periphery). They considered clinically significant moderate differences, those that were higher or equal to 10 and important differences those that were higher or equal to 20. Worst baseline functional scores were observed for global QoL (62.9 ± 1.1), PF (61.8 ± 1.1), and role functioning (63.5 ± 1.5). They reported no statistically or clinically significant worsening of any of the QoL functioning or symptom scores over time except for physical functioning with a statistically significant decrease at 18 and 24 months (5.7 and 5.6 points, respectively, *P* < 0,01). With a median overall survival of 40 months, they found that baseline QLQ-C30 PF score was a strong independent predictor of survival (HR 1.44, 95% confidence interval [CI] 1.01-2.05; *p* = 0.045), as well as performance status (*P* = 0.047), Charlson comorbidity index (*P* = 0.008), and pulmonary function (*P* = 0.027).

Van der Voort van Zip *et al*. [34] published in 2010 a prospective study in 39 patients with histological confirmation of early stage NSCLC. EORTC QLQ-C30 and the QLQ LC13 lung cancer-specific questionnaires were used. Tests were done at baseline, at 3 weeks and 2, 4, 6, 9, and 12 months after treatment. Patients whose tumor progressed were excluded to avoid bias in result interpretation. The percentage of compliance with the tests during follow-up was 95% or higher. With a median follow-up of 17 months, they did not observe a deterioration in QoL nor in respiratory symptoms. Strangely, dyspnea worsening was detected at 6 months post-treatment, but it posteriorly got back to basal levels. The only significant change observed was an improvement in scores for emotional functioning (*P* = 0.02).

Importantly, just one open label randomized controlled prospective Phase III clinical trial was published on the topic. The ROSEL study was published by Louie *et al*. in 2015 [22] and it was a comparison between SBRT treatment and surgery in Stage I NSCLC. Primary objectives were local and regional control, treatment costs and quality of live outcomes measured with EORTC QLQ-C30 plus LC13. It was prematurely closed due to lack of recruitment. Eleven patients were managed to be treated in each treatment group. Clinical significance in this case was also meaningful when QoL scores differed in more than 10 points. Regarding result on QoL, global health scores in the univariate analysis were significantly worse in the surgery group compared to the SBRT group (HR 0.19, *P* = 0.038).

Widder *et al*. [28] published a prospective observational study where 202 patients who were medically inoperable and who had T1-T2 tumors, went to receive SBRT (60 Gy in 3, 5, or 8 fractions). As a control group they had 27 patients that were treated with 3DCRT (70 Gy in 35 fractions). QoL scores were collected using the EORTC QLQ-C30 and the LC-13 for dyspnea. In agreement with other previous investigators, they defined clinically meaningful changes as those with 10 or more-point difference. With a median follow-up of 13 months, global QoL and PF remained stable except in patients with a high Charlson comorbidity index (CCI>3) where a deterioration of PF was detected (-2.5-0.2, *P* = 0.02). Dyspnea symptom had a statistically significant increase of 3.2 (95% CI: 1.0–5.3; *P* < 0.01) but did not meet the criteria to be clinically meaningful. Global QoL scores did not differ during follow-up between groups, although a significant decline in PF (*P* < 0.01) and a tendency for higher scores in dyspnea were seen after 3DCRT versus SBRT.

Finally, Adebahr *et al*. [29] published a prospective monocenter Phase II STRIPE trial that included 100 patients with lung lesions of 5 or less centimeters treated with SBRT. Out of the 100 patients, 56 were non-surgical candidates with early stage NSCLC and 44 were oligometastatic lesions in the lung (≤2) with a controlled primary tumor. The primary objective of the study was the early impact of the treatment on patient QoL. They employed EORTC QLQ-C30 questionnaires for this objective. Once again, clinically relevant changes in QoL were defined as those with a change in 10 or more points per item. Tests answered at baseline demonstrated lowest punctuation for global QoL scores (52.4±21.3), EF (59.3 ± 26.8), and role function (49.6 ± 31.2). Most severe symptoms were dyspnea (49.1 ± 33.5 on the QoL C30 and 39.7 ± 24.0 on the QoL LC13), coughing (39.5 ± 20.7), and fatigue (46 ± 27.9). Early impact of treatment was measured 7 weeks after SBRT and clinically meaningful changes in symptoms were not detected, although an emotional function change was detected from baseline 7.7 ± 21.4 (*P* = 0.002) as well as a small improvement in global QoL/GHS (mean difference [MD]: 4.4 ± 20.3; p = 0.034). In the subgroup analysis, patients with worse basal scores in QoL/GHS (below 50) experimented a clinically significant improvement in comparison to those who had basal good scores (above 50) that remained stable. Similarly, patients with worse dyspnea scores at baseline (40 points) experimented a clinical improvement in EF, fatigue, and dyspnea post-SBRT. In a posterior analysis of the results published by Nestle *et al*. [31], 2-year follow-up showed no difference in QoL/GHS as well as no difference in symptoms, except for a transient worsening in pain scores. A clinically meaningful change was detected at 2 years for dyspnea in the EORTC QLQ-C30 (↑10.2) scale, but it did not match a clinically meaningful change for the LC-13 test. Once again, subgroup analysis confirmed that patients with lower QoL/GHS records at baseline do better after SBRT treatment (*P* < 0.001) and have dyspnea improvement in the LC-13 module (*P* < 0.001) and fatigue improvement (*P* < 0.01). Patients with a higher PF improvement were those that had a KI>80% and a Charlson of CCI>7.

#### 3.2.1. Reviews

In 2016, Chen *et al*. [35] published a systematic review on patient-reported health-related QoL (HRQOL) after SBRT treatment for early stage NSCLC. This review included nine articles that met selection criteria, all of them prospective in design. Overall, studies had few changes as far as HRQOL scores are concerned, although two out of nine studies had isolated clinically and statistically significant worsening in symptoms: one study in dyspnea and one study in fatigue. Authors conclude that SBRT in early stage NSCLC is safe and has a minimum impact on health-related QoL.

In 2018, two reviews were published: one that aimed at comparing SBRT with minimal invasive surgery [14] and one that aimed at reviewing SBRT toxicity and patient reported QoL [16].

The first was written by Pompili *et al*. [14], and its primary objective was the comparison of the QoL impact between SBRT and minimally invasive thoracic surgery. Sixteen studies were included for review and only one prospective randomized controlled study was detected written by author Louie *et al*. [22]. The other 15 articles separately investigated the effect of stereotactic ablative body radiotherapy or VATS lobectomy on QoL. General results for the surgical group were that HRQOLT at 3 months had a clear deterioration and that these values improved at 1-year follow-up going back to baseline scores. In the SBRT group on the contrary, HRQOLT values remained unchanged during the 1^st^ year follow-up.

The second and last were the review by Donovan *et al*. [16], where SBRT toxicity and HRQOL results including a variety of QoL tests were explored. Article search strategy is not outlined but three conclusions are drawn with evaluated literature. First, they conclude that SBRT is a well-tolerated technique with a similar local control to that of surgery. Second, they highlight the much smaller impact on toxicity by SBRT compared to surgery, both acute and chronic. And third, although SBRT population has many more comorbidities and worse pulmonary function at baseline, extracted information in QoL in the different studies gives a sense that HRQOL scores after SBRT are comparable if not improved to those of surgery. Finally, they define an appropriate candidate for SBRT as the one with either moderate to severe COPD, with comorbidities related to postsurgical complications, or an elderly susceptible to physical and functional decline following surgery.

## 4. Discussion

Early stage lung cancer is a curable disease and thus, patients treated with either surgery or radiosurgery can have long tumor-specific survivals. This characteristic makes adding HRQOLT of utmost importance in this population. In addition, patients diagnosed with ES-NSCLC are very frequently smokers or past-smokers with COPD, have cardiovascular comorbidities and are fragile in nature due to their advanced age. Both, the long tumor-specific survival and patient frailty are strong factors to need a careful examination of patient QoL before treatment selection or discussion.

Scientific evidence in ES-NSCLC favors treatment with lobectomy surgery (IB). This surgery has evolved to be minimally invasive to avoid postoperative complications and related long hospitalizations. Post-operative lung function depends on four factors: resected lung volume, baseline respiratory function, the existence of previous lung disease, and the resected lung lobe. Estimated lung function deterioration after lobectomy with VATS is of 5% for each resected segment [36]. In COPD patients, lobectomy can represent a median loss of FEV1 around 0.11L (range -0.33-0.09L) [37]. In addition, recent studies reflect that 1 year is needed after surgery for pulmonary function to recover, and that there are no clear differences in lung function deterioration between VATS and thoracotomy in the long run [38]. Post-SBRT dyspnea, as a surrogate of lung function deterioration, has been described in some studies. However, some authors argue that it is the clinical natural course of COPD [39] and therefore, not a symptom related to SBRT treatment. Concerning lung function, prospective studies in SBRT population did not find statistically significant changes at 3 years post-treatment [40].

Nevertheless, whether SBRT is equivalent to VATS is not known, as past randomized controlled studies comparing HRQOLT in SBRT and surgery were closed due to insufficient patient accrual (ROSEL and STARS) [41,42]. A pooled analysis of ROSEL and STARS trials suggested a better 3-year survival with SBRT in comparison to surgery [43], although these results must be interpreted with caution as they represent a small sample population. In the same line, a published meta-analysis on retrospective data comparing the effectiveness of SBRT and surgical resection in ES-NSCLC found that 3-year survival of sublobar resection (SLR) and SBRT was comparable [44]. The answer to whether SBRT is comparable to surgery in operable patients will hopefully come with the results of the ongoing randomized controlled trials (VIOLET and VALOR).

This review attempted to update health related QoL data on patients treated with VATS or SBRT in ES-NSCLC. Studies that have been selected to measure QoL outcomes mix patients that are medically operable with those that are not. Similarly, some operable patients refuse surgery and are therefore treated with SBRT. Altogether, it seems important to point out that the comparison between these different populations is a selection bias present in this review, and that this bias has been previously mentioned in the field of ES-NSCLC treatment choice [45,46].

Out of the 23 articles selected for review, 14 evaluated QoL in patients treated with SBRT and five in patients treated with VATS. Just one study with 22 patients was found to be designed specifically to compare surgery to SBRT [22]. Median age of the studies in the SBRT group was much higher than the median age in the VATS group, and generally was accompanied by mayor comorbidities.

In the retrospective study by Schwartz [21], SEER database was used to compare SBRT to surgery. SBRT group of patients had many more cardiovascular comorbidities, and specifically, heart pathology was the most frequent. In contrast, SBRT group did not show a higher PCS deterioration to the surgical group, nor were there differences in MCS. In the same way, Adebahr *et al*. study [29] concluded that patients with baseline low scores on global health were those who benefited most from treatment with SBRT.

Globally, studies in the VATS and SBRT groups had different data collection designs. 12 of the SBRT studies had three data collection time-points post-SBRT and only Rutkowski *et al*. study [32] had two data collection time-points post-SBRT. When we analyze the follow-up period of each study, the SBRT study by Ubels *et al*. [33] had a 5-year follow-up, which was the longest we found throughout the included articles. In contrast, the longest follow-up found in the VATS studies was 1 year. In conclusion, we found that works from the SBRT group were more robust as far as follow-up and QoL data are concerned.

When therapy impact on physical score was evaluated, 2 of 13 studies in the SBRT group did not detect statistically significant changes [34]. Although having a short follow-up, Rutkowski’s group [32] on the contrary, detected an improvement after SBRT treatment in GH and PF that were statistically significant. Ubels *et al*. study [33], with the longest follow-up, showed a significant change in PF, RF, and CF with a gradual improvement detected by QLQ-C30 tests but not by LC13. On the other hand, in the VATS group, no statistically significant improvement was seen on the physical scores.

Throughout the studies and the reviews, different factors were identified to help determine the patients that would benefit from one or other treatment. Nestle *et al*. [31] showed, for example, that patients with lower ECOG scores before treatment were the ones who would benefit most from SBRT instead of surgery as far as QoL data on GHS is concerned. These results were previously confirmed by Adebahr *et al*. [29]. We found just one study by Lagerwaard *et al*. that detected a deterioration on PFS scores in the SBRT group. In the VATS studies in contrast, four out of the five studies demonstrated a deterioration in PCS QoL scores.

MCS in the Schwartz *et al*. [21] study that compared QoL data in SBRT and surgery did not change in the SBRT cohort but had a statistically significant deterioration in the surgery group. This post-surgery MCS deterioration is not confirmed in Anami *et al*. study [18], probably due to the fact that they used different questionnaires.

Global health status (GH) is improved post-treatment in three out of 13 SBRT studies and in none of the VATS studies. It is worth mentioning that in the study by Nestle *et al*. [31] patients with baseline GH scores below 50 were those that, after SBRT treatment, had a GH score improvement.

## 5. Conclusions

Given the heterogeneity of published studies and the absence of well-designed clinical trials with sufficient patients treated in each arm, making a recommendation in terms of QoL impact between VATS and SBRT is not prudent. According to the data published, patients have a minimum to null impact on QoL after SBRT despite having a worse physical function at baseline. Therefore, those who have cardiovascular comorbidities, ECOG 1-2 scores or that are fragile, could be the most to benefit from this treatment. On the other hand, studies with VATS treatment had a shorter follow-up with fewer QoL data collected than SBRT studies, but did demonstrate a QoL deterioration after treatment in the different QoL items evaluated. To make an accurate patient selection for either VATS or SBRT treatment in ES-NSCLC, we recommend a QoL assessment, and geriatric assessment when appropriate, before treatment selection or discussion. Results from prospective randomized studies that are ongoing will bring the necessary scientific evidence to make further recommendations in the future.

### Conflict of Interest

The authors declare that there are no known conflicts of interest associated.
